# Ubiquitin Activating Enzyme UBA6 Regulates Th1 and Tc1 Cell Differentiation

**DOI:** 10.3390/cells11010105

**Published:** 2021-12-29

**Authors:** Ji Yeon Lee, Eun-Koung An, Juyoung Hwang, Jun-O. Jin, Peter C. W. Lee

**Affiliations:** 1 Division of Rheumatology, Department of Medicine, Seoul St. Mary’s Hospital, Catholic University, Seoul 06591, Korea; 2 Department of Medical Biotechnology, Yeungnam University, Gyeongsan 38541, Korea; eunkoungan@yu.ac.kr (E.-K.A.); jyhwang5@yu.ac.kr (J.H.); 3 Research Institute of Cell Culture, Yeungnam University, Gyeongsan 38541, Korea; 4 Department of Biomedical Sciences, University of Ulsan College of Medicine, Asan Medical Center, Seoul 05505, Korea; 5 Lung Cancer Research Center, University of Ulsan College of Medicine, Asan Medical Center, Seoul 05505, Korea

**Keywords:** UBA6, T cell, ubiquitin, differentiation, multiorgan inflammation

## Abstract

Ubiquitination is a crucial mechanism in regulating the immune response, setting the balance between immunity and tolerance. Here, we investigated the function of a poorly understood alternative branch of the ubiquitin-activating E1 enzyme UBA6 in activating immune cells. UBA6 expression levels were elevated in T cells by toll-like receptor agonists and anti-CD3/28 antibody stimulation, but not in dendritic cells, macrophages, B cells, and natural killer cells. Additionally, we generated T cell-specific UBA6-deficient mice and found that UBA6-deficient CD4 and CD8 T cells elevated the production of interferon-gamma (IFN-γ). Moreover, the transfer of UBA6-deficient CD4 and CD8 T cells in RAG1-knockout mice exacerbated the development of multi-organ inflammation compared with control CD4 and CD8 T cell transfer. In human peripheral blood CD4 and CD8 T cells, basal levels of UBA6 in lupus patients presented much lower than those in healthy controls. Moreover, the IFN-γ production efficiency of CD4 and CD8 T cells was negatively correlated to UBA6 levels in patients with lupus. Finally, we found that the function of UBA6 was mediated by destabilization of IκBα degradation, thereby increasing NF-κB p65 activation in the T cells. Our study identifies UBA6 as a critical regulator of IFN-γ production in T cells by modulating the NF-κB p65 activation pathway.

## 1. Introduction

Protein ubiquitination is a reversible covalent modification that regulates the activity, localization, and stability of target proteins. While generally considered a terminate signal for proteasomal degradation, ubiquitination is also appreciated for its functions in protein modulation, including receptor down-modulation, protein–protein interactions, and gene transcription [1]. Protein ubiquitination has also emerged as a critical mechanism of immune response regulation, including lymphocyte development and activation, intracellular signal transduction, antigen presentation, and immune evasion [2,3]. Recent work has shown that various ubiquitin (Ub) ligases help to prevent the immune system from attacking self-tissues, implicating the dysfunction of Ub in autoimmune diseases [2,4].

Ub-activating (E1) enzymes catalyze the first step in the ubiquitination cascade, which results in the addition of Ub and other Ub-like proteins to target proteins. Besides UBE1 (Ub-activating enzyme 1 [UBA1]), Ub-activating enzyme 6 (UBA6) is an alternative enzyme for Ub activation [5,6,7,8]. Mice lacking UBA6 died at E5.5 of embryonic development, indicating the importance of UBA6 during embryogenesis [5,9]. The physiological functions of UBA6 remain poorly understood; however, we have previously reported that UBA6 is required for the viability of mouse embryo fibroblasts (MEFs), and loss of neuronal UBA6 resulted in abnormal hippocampal and amygdala development [9,10,11,12]. UBA6 is abundantly expressed in many organs, including the thymus and peripheral lymphoid organs, raising the question of UBA6 function in T cells. The distinguishing feature of UBA6 from UBA1 is that it is required for activation of the E2-conjugating enzyme USE1 (also referred to as UBE2Z), a lysine-48 chain linkage-specific E2 [10]. Of the approximately two dozen E2 enzymes encoded by the human genome, USE1 relies solely on UBA6 for charging [7,10]. Moreover, to date, USE1 has been shown to function together with UBR1, UBR2, and UBR3 [10,13]. These E3s constitute primary members of the N-end rule class of E3s, and in this context, the UBA6–USE1–UBR pathway has been shown to control the turnover of RGS proteins [10]. However, this pathway likely controls the turnover of additional proteins through other E3 ligases.

Ub has a central role in the regulation of several pathways leading to the activation of NF-κB, which is vital for both innate and adaptive immunity [2]. NF-κB is necessary for T cell activation, survival, and proliferation, as well as effective immune responses [2,14]. Given the established roles of Ub and NF-κB in T cell responses, we hypothesized that UBA6 could be involved in the activation and function of immune cells. We induced immune cell activation to test this hypothesis and found that UBA6 levels were elevated in T cells only. Subsequently, we generated T cell-specific conditional UBA6-deficient mice under the control of the proximal lck promoter and evaluated the function of UBA6 in T cell activation. We found that deletion of UBA6 in T cells greatly increased differentiation and proliferation in immune stimulation. This function of UBA6 was mediated by destabilized IκB degradation, resulting in increased activation of NF-κB p65. These data indicated that alteration of the UBA6 pathway in T cells leads to defects in T cell response homeostasis and demonstrates a novel role for UBA6 in controlling T cell receptor (TCR) signal transduction pathways.

## 2. Materials and Methods

### 2.1. Mice

UBA6^flox/flox^ mice have been previously described [9,12]. Lck-Cre mice and RAG1^−/−^ mice were obtained from the Jackson Laboratory and C57BL6 mice were purchased from Hychang Science (Daegu, Korea). Mice were then bred at the animal facilities of the Asan Institute for Life Sciences, University of Ulsan College of Medicine, and Yeungnam University animal facility. The animal procedures were approved by the Institutional Ethics Committee and Institutional Animal Care Committee of the University of Ulsan College of Medicine (2019-12-218, 2019-12-316) and Yeungnam University (2020-030). All animal studies were conducted in a double-blind manner by separating animal breeding and tissue analysis. Mouse experiments were repeated twice, and the numbers of animals in the experimental groups are described in detail in the Figures. 

### 2.2. Human Blood Samples

This study was conducted according to the principles of the Declaration of Helsinki. Thirty cc of whole peripheral blood was obtained from patients with systemic lupus erythematosus (SLE) and healthy controls at Seoul St. Mary’s Hospital. The enrollment criteria for patients with lupus included those age 20–60 years who had been followed in the rheumatology clinic for their diagnosis of SLE per the American College of Rheumatology criteria [15]. The enrollment criteria for healthy controls included males or females aged between 20 to 60 years without autoimmune or inflammatory diseases. We excluded patients with any serious systemic illness, including infection and cancer. Written informed consent was obtained from all patients, and the Institutional Review Board of Seoul St. Mary’s Hospital, in Seoul, Korea, approved the study (IRB number: KC20TISI0491). 

### 2.3. Antibodies and Reagents

Functional grade anti-CD3 (145-2C11), anti-CD28 (37.51), anti-IL-4 (11B11), and anti-IFN-γ (H22) were purchased from BioXCell (West Lebanon, NH, USA). Fluorescently labeled anti-B220 (RA3-6B2), anti-CD3 (17A2), anti-CD4 (RM4-5), anti-CD8 (53-6.7), anti-CD11b (M1/70), anti-CD11c (N418), anti-CD25 (PC61), anti-NK1.1 (PK136), and anti-TCR-β (H57-597) were purchased from BioLegend (San Diego, CA, USA). Fluorescently labeled anti-phospho-c-Jun (Ser63) (KM-1) was obtained from Santa Cruz Biotechnology (Santa Cruz, CA, USA), and anti-ATF4 (D4B8), anti-GRP78, anti-PERK (C33E10), anti-phospho-PERK (16F8), anti-eIF2a (D7D3), anti-phospho-eIF2a (Ser51) (D9G8), anti-phospho-p65 (Ser536) (93H1), anti-IκBα, anti-phospho-IκBα (Ser32), and anti-phospho-Erk1/2 (Thr202/Tyr204) (D13.14.4E) were provided by Cell Signaling Technologies (Danvers, MA, USA). FAT10, UBA6, and USE1 antibody were described previously [9,10,13]. The recombinant murine IL-1β (rmIL-1β), IL4, IL-6, IL-12p70, IL-23, and TGF-β1 were purchased from BioLegend (San Diego, CA, USA). 

### 2.4. Flow Cytometry Analysis

Single-cell suspensions from the thymus, lymph node, spleen, lung, or liver were prepared and resuspended in staining buffer (PBS containing 1% FBS and 2 mM EDTA) and stained with the indicated antibodies (Abs). Cells were incubated with PMA plus ionomycin for 4 h, and GolgiStop (BD Bioscience, San Jose, CA, USA) was added 2 h before harvesting the cells to detect cytokines. To detect transcription factors (phospho-p65 and phospho-c-Jun), we followed the manufacturer’s protocol from eBioscience (San Diego, CA, USA). Data were acquired by NovoCyte (ACEA Biosciences Inc., San Diego, CA, USA) and analyzed with NovoExpress (ACEA Biosciences Inc., San Diego, CA, USA).

### 2.5. T Cell Isolation and Activation

Naïve-enriched CD4 or CD8 T cells were purified by negative selection using CD4^+^ or CD8^+^ T cells isolation kits (Miltenyi Biotec, Bergisch Gladbach, Germany). For in vitro studies, isolated CD4 or CD8 T cells (1 × 10^6^ per well in a 24-well plate) were stimulated with a plate coated in 5 μg/mL of anti-CD3 mAb plus anti-CD28 mAb for 3 days. Splenocytes were stimulated with soluble anti-CD3 (1 μg/mL) and anti-CD28 (1 μg/mL) for 3 days to differentiate T cells. Cytokines or cytokine-blockade agents were added in culture as indicated. IL-1β, IL-4 IL-6, IL-12, IL-23, and TFG-β were used at concentrations of 10 ng/mL, 10 ng/mL, 50 ng/mL, 50 ng/mL, 20 ng/mL, and 20 ng/mL, respectively. Anti-IL-4 and anti-IFN-γ, as well as their respective control IgGs, were all used at 10 μg/mL. All cell cultures were maintained in RPMI 1640 (Invitrogen, Carlsbad, CA, USA) supplemented with 10% FBS, 2 mM L-glutamine, 10 mM HEPES, 1% penicillin/streptomycin, and 50 μM β-mercaptoethanol. 

### 2.6. In Vivo T Cell Stimulation by Anti-CD3 Antibody

C57BL/6 mice were injected with 20 μg of anti-CD3 Abs. Twenty-four h after injection, mice were euthanized and splenocytes were isolated and restimulated in vitro for 4 h with phorbol 12-myristate 13-acetate (50 ng/mL) and ionomycin (1 μM; both from Calbiochem, La Jolla, CA), with addition of monensin (eBioscience) during the final 2 h. Cells were then stained for intracellular cytokines and surface markers using the intracellular cytokine staining kit (BioLegend).

### 2.7. Induction of Multi-Organ Inflammation in RAG1-Knock out Mice

The CD3^+^CD25^−^CD122^−^ T cells from C57B/6 or UBA6D mice splenocytes were isolated using a mouse pan T cell isolation kit (Miltenyi Biotec) with biotin-conjugated anti-CD25 and anti-CD122 Abs. The CD3^+^CD25^−^CD122^−^ cells (1 × 10^6^/100 μL) were transferred into 6–8-week-old RAG1-KO mice via intravenous injection. After 2 weeks, the mice received 20 μg of polyinosinic:polycytidylic acid (poly I:C) three times per week for an additional 2 weeks. Mice were then euthanized, and organs were collected for analysis.

### 2.8. Preparation of Lung and Liver Cell Suspension

Mice lungs and liver were dissected into small fragments, placed in a grinder, and processed in a tissue homogenizer. Tissue homogenates were filtered through a 100 μm nylon mesh, washed twice in PBS, and resuspended in culture medium.

### 2.9. Histology and Immunofluorescence Staining

Lung, liver, and colon samples were fixed with 4% paraformaldehyde and embedded in paraffin. The tissues were then sectioned into 5 μm slices and stained with hematoxylin and eosin (H&E) after deparaffination and rehydration. 

### 2.10. Human T Cell Analysis

Peripheral blood mononuclear cells (PBMCs) were prepared from the whole blood of healthy or SLE donors using a density gradient centrifugation method with Histopaque-1077 (Sigma-Aldrich, St. Louis, MO, USA). Cells were then stained with anti-CD3 (OKT3), anti-CD4 (OKT4), and anti-CD8 (SK1). Some methods required that CD4 or CD8 T cells be isolated from PBMCs using a human CD4 or CD8 T cell isolation kit (Miltenyi Biotec). The cells were then stimulated with pre-coated anti-CD3/28 mAb for 3 days. Intracellular production levels of IFN-γ were measured as described above. 

### 2.11. Immunoblotting

Whole-cell lysates were prepared in ice-cold RIPA lysis buffer containing a protease inhibitor cocktail (Roche, Basel, Switzerland) and a phosphatase inhibitor cocktail (Pierce). Protein concentration was measured using the BCA method (Pierce). After boiling in loading buffer, lysates were separated on SDS-PAGE and analyzed by western blotting using the following antibodies: (phospho-IκBα, IκBα), ATF4, (phosphor-PERK, PERK), (phosphor-eIF2, eIF2), GRP78, UBA6, USE1, and Fat10. The membrane was stripped and reprobed with α-tubulin Ab (Abcam, Cambridge, UK) or UBA6-specific polyclonal Ab, generated as described previously [7]. Horseradish peroxidase (HRP)-conjugated anti-rabbit or anti-mouse secondary Abs (GE Health, Chicago, Illinois, USA) and ECL (GE Health) were used to detect bound Abs. 

### 2.12. Quantitative PCR

RNA was isolated by Trizol or easy-BLUE extraction according to the manufacturer’s instructions (Invitrogen and iNtRON Biotechnology, Seongnam, Korea). CDNA was synthesized using SuperScript II Reverse Transcriptase (Invitrogen and iNtRON Biotechnology) and subjected to quantitative real-time PCR using SYBR Green I Master Mix on a CFX Connect Real-Time PCR system (Bio-Rad, CA, USA). Each value was normalized to human β-actin or GAPDH. The specific primers used for PCR amplification were previously described [15].

### 2.13. Nuclear NF-κB p65 Activation Assay

According to the manufacturer’s protocol, the nuclear NF-κB p65 activity was quantified using an ELISA-based TransAM NF-κB p65 kit (Active Motif, Carlsbad, CA, USA). In brief, whole cell protein extracts (10 µg per well) were added to a 96-well plate containing an immobilized oligonucleotide with an NF-κB element for 1 h incubation at room temperature, during which activated NF-κB p65 could bind specifically to the oligonucleotide. NF-κB p65 Ab (100 µL, at 1:1000 dilution) was then added to each well for 1 h followed by 100 µL of anti-rabbit HRP-conjugated Ab (1:1000 dilution) for 1 h. After washing three times in wash buffer, developing solution (100 µL) was added for up to 15 min, and the colorimetric reaction was stopped after 5 min. The NF-κB p65 activity was determined by reading absorbance on a spectrophotometer (Molecular Devices, CA, USA) at 450 nm with a reference wavelength of 655 nm. 

### 2.14. Statistical Analysis

All statistical analyses were performed using Prism software, version 5 (GraphPad, San Diego, CA, USA). Results are presented as mean ± SEM and a one- or two-way ANOVA (Tukey multiple comparison test) were used for analysis of the data sets. Asterisks denote the level of significance (* *p* < 0.05, ** *p* < 0.01), and a *p*-value of < 0.05 was considered statistically significant.

## 3. Results

### 3.1. Expression Levels of UBA6 in Immune Cells in Response to Stimulation

**Figure 1:**
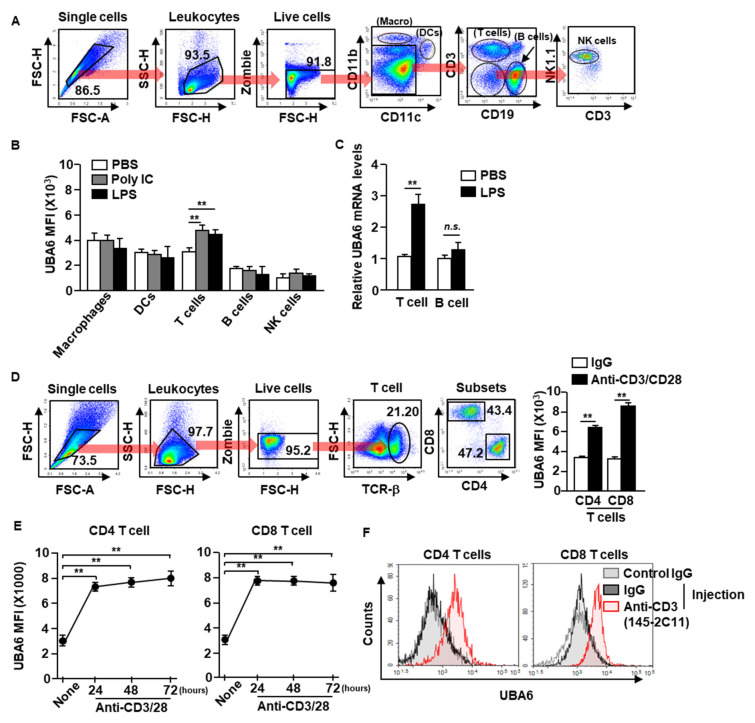
Elevation of UBA6 expression in stimulated T cells. C57BL/6 mice were administered intraperitoneal injection with 2 mg/kg of poly IC and 0.1 mg/kg of LPS. Twenty-four h after injection, spleens were collected and UBA6 expression levels in immune cells were measured. (**A**) Gating strategy of immune cells in the spleen was observed. (**B**) Mean fluorescence intensity (MFI) of UBA6 in the indicated immune cells (*n* = 6 mice, two-way ANOVA, mean ± SEM, ** p < 0.01). (**C**) UBA6 mRNA expression levels in isolated T and B cells 24 h after stimulation with LPS (*n* = 4 mice, two-way ANOVA, mean ± SEM). (**D**) Splenocytes were incubated with anti-CD3/28 Ab for 24 h. Gating strategy of CD4 and CD8 T cells (left panel) and MFI of UBA6 in CD4 and CD8 T cells (right panel, *n* = 6 mice, two-way ANOVA, mean ± SEM, ** p < 0.01). (**E**) Time-dependent expression levels of UBA6 in CD4 or CD8 T cells were shown after stimulation with anti-CD3/28 Abs (*n* = 6 mice, two-way ANOVA, mean ± SEM, ** p < 0.01). (F) C57BL/6 mice were injected *i.p.* with stimulatory anti-CD3 Ab and measured UBA6 levels in splenic CD4 and CD8 T cells 24 h after injection.

To define UBA6 function in immune cells, we first examined the alteration of UBA6 expression levels in immune cells after stimulation with toll-like receptor agonist poly I:C or lipopolysaccharide (LPS). C57BL/6 mice were treated intraperitoneally with poly I:C or LPS, and UBA6 expression levels in the immune cells were analyzed 24 h after treatment. Macrophages, dendritic cells (DCs), natural killer (NK) cells, T cells, and B cells in the spleen were defined by specific marker expression as shown in Figure 1A. The intracellular expression levels of UBA6 increased significantly in T cells after poly I:C or LPS stimulation compared with PBS-treated controls, and expression levels did not change in other immune cells (Figure 1B). In addition, mRNA levels of UBA6 levels in isolated T cells were also significantly upregulated by LPS stimulation, whereas it was not increased in the isolated B cells (Figure 1C). To determine the stimulation dependent UBA6 levels in T cells, splenocytes were incubated with anti-CD3/CD28 Abs, which caused considerable upregulation of UBA6 levels (Figure 1D). In addition, these upregulated UBA6 levels were maintained for 3 days in CD4 and CD8 T cells (Figure 1E). UBA6 expression in both CD4 and CD8 T cells in the spleen was also substantially elevated by administering stimulatory anti-CD3 Ab to mice in vivo (Figure 1F). Thus, these data indicated that UBA6 may contribute to the activation of CD4 or CD8 T cells in response to stimulation.

### 3.2. UBA6 Is a Negative Regulator of IFN-γ Production in CD4 and CD8 T Cells

**Figure 2:**
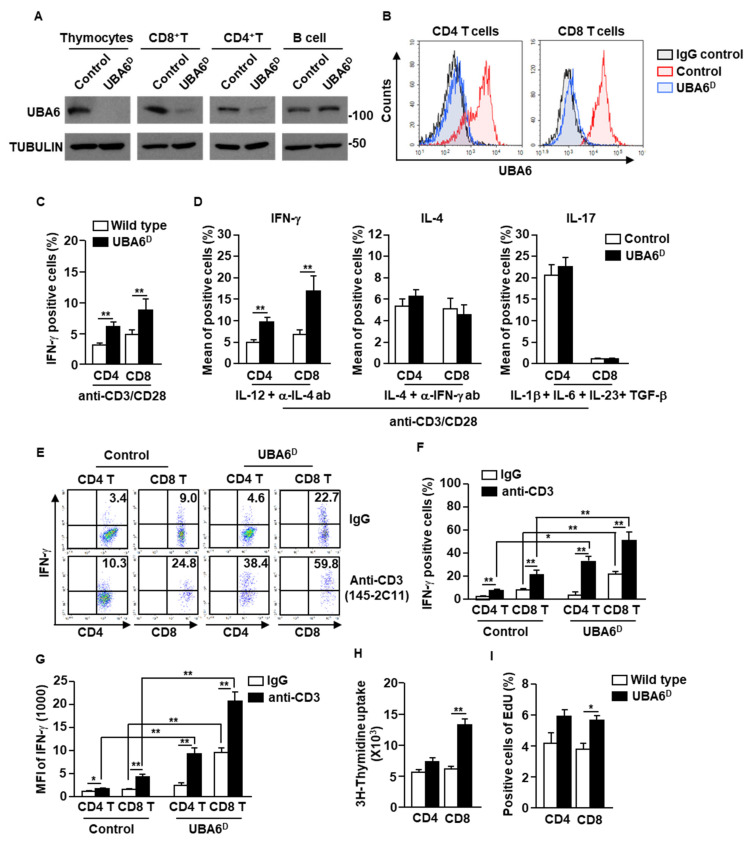
Conditional deletion of UBA6 increased production of IFN-γ in CD4 and CD8 T cells. Total thymocytes, peripheral T cells, and CD19^+^ B cells were isolated from the thymus and spleen of control and UBA6^D^ 6 week old mice. (**A**) Cell lysates were subjected to immunoblot with anti-UBA6 and anti-tubulin. (**B**) Expression levels of UBA6 in UBA6^D^ CD4 and CD8 T cells were shown. (**C**) Intracellular producing levels of IFN-γ were measured after stimulation with anti-CD3/28 Ab (*n* = 6 mice, two-way ANOVA, mean ± SEM, ** p < 0.01). (**D**) Intracellular production levels of indicated cytokines were analyzed after treatment with cytokine and mAbs (*n* = 6, two-way ANOVA, mean SEM, ** p < 0.01). (**E**) Splenic CD4 and CD8 T cells were analyzed for intracellular production levels of IFN-γ 24 h after injection with stimulatory anti-CD3 Ab. (**F**) Mean IFN-γ-producing cells. (**G**) MFI of IFN-γ levels were shown (*n* = 6 mice, two-way ANOVA, mean ± SEM, * p < 0.05, ** *p* < 0.01). (**H**) Cell proliferation was measured by ^3^H-thymidine uptake after stimulation with anti-CD3/28 Ab (*n* = 6 mice, two-way ANOVA, mean ± SEM, ** p < 0.01). (**I**) Cell proliferation in the in vivo splenic CD4 and CD8 T cells were measured by Edu uptake (*n* = 6 mice, two-way ANOVA, mean ± SEM, * p < 0.05).

To examine the specific function of UBA6 in T cells, we generated Lck-cre UBA6^flox/flox^ mice. For simplicity, the Lck-Cre/UBA6^flox/flox^ mice with deletion of UBA6 alleles are referred to herein as UBA6D, whereas the UBA6^flox/flox^ control mice are referred to as UBA6FL. We confirmed that UBA6 deletion was specific in T cells (thymocytes, CD4^+^, and CD8^+^), but did not occur in CD19^+^ B cells by immunoblot analysis of UBA6 (Figure 2A). Moreover, intracellular expression levels of UBA6 also decreased remarkably in UBA^D^ T cells compared with T cells in control mice (Figure 2B). UBA6^D^ mice were born at the expected Mendelian ratios and appeared normal at a young age. In addition, unlike Ub E3 ligase Cbl or Cbl-b KO mice [4,16,17], UBA6^D^ mice did not develop features of spontaneous inflammation or autoimmunity by 6–8 months of age (our experimental observation period). 

The isolated CD4 and CD8 T cells were then stimulated with anti-CD3/28 Abs for 3 days. Compared with control T cells, UBAD T cells greatly increased intracellular production of IFN-γ (Figure 2C and Supplementary Figure S1A). In the T cell differentiation assay using cytokine and Ab combination, Th1 and Tc1 differentiation was significantly elevated in UBA6^D^ T cells compared with control T cells. In contrast, the efficacy of Th2 and Th17 differentiation did not differ between UBA6D and control T cells (Figure 2D and Supplementary S1B). In addition, stimulatory anti-CD3 Ab injection in UBA6^D^ mice promoted much greater intracellular IFN-γ production in both CD4 and CD8 T cells than in control mice T cells (Figure 2E–G). We further examined the proliferation of T cells in control and UBA6^D^ mice and found that the UBA6^D^ T cells increased proliferation capacities in mice in vitro (Figure 2H) and in vivo (Figure 2I) in response to anti-CD3 Abs. Thus, these data indicated that UBA6 controls the production of IFN-γ in T cells and proliferation of T cells in response to anti-CD3/28 Ab stimulation. 

### 3.3. Exacerbation of Multi-Organ Inflammation in RAG1-Knockout Mice by UBA6^D^ T Cell Transfer

**Figure 3:**
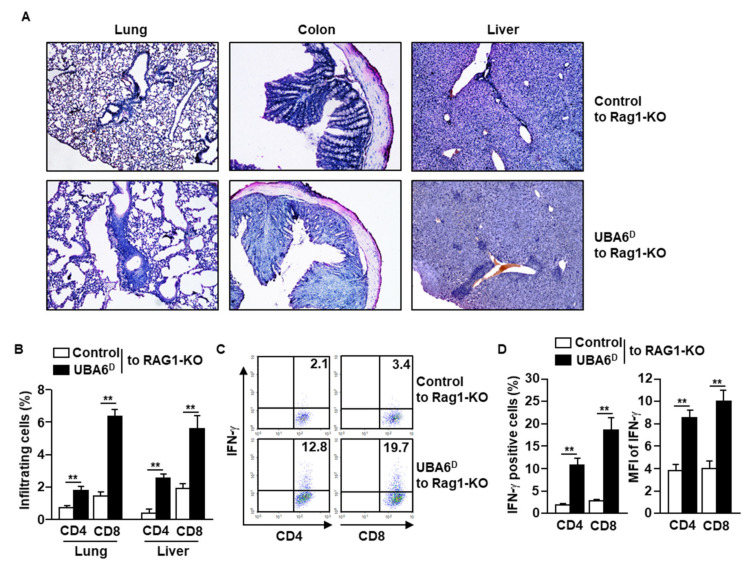
RAG1-KO mice transferred with UBA6^D^ T cells developed multi-organ inflammation. CD3^+^CD25^−^CD122^−^ cells were isolated from control or UBA6^D^ mice and transferred into RAG1-KO mice. (**A**) Leukocyte infiltration in peripheral tissue was analyzed by H&E staining. (**B**) Lung- and liver-infiltrated CD4 and CD8 T cells were analyzed with flow cytometry (*n* = 6 mice, two-way ANOVA, mean ± SEM, ** p < 0.01). (**C**) Intracellular IFN-γ producing levels in CD4 and CD8 T cells were measured. (**D**) Mean positive cells (left panel) and MFI of IFN-γ expression (right panel) were shown (*n* = 6 mice, two-way ANOVA, mean ± SEM, ** p < 0.01).

Since UBA6^D^ T cells are hyperactivated to produce intracellular IFN-γ in response to anti-CD3/28 Abs, we next examined whether UBA6^D^ T cells are involved in the development of multi-organ inflammation. As indicated in the infiltration of immune cells in the lung, colon, and liver, RAG1-KO mice with UBA6^D^ T cell transfer had exacerbated multi-organ inflammation compared with RAG1-KO mice with control T cell transfer (Figure 3A). Consistent with the H&E staining data, lung and liver infiltrated UBA6^D^ T cell levels were much higher than those of infiltrated control T cells (Figure 3B). Furthermore, the intracellular production levels of IFN-γ also increased significantly in the UBA6^D^ T cell transferred mice than in control T cell transferred mice (Figure 3C,D). These data therefore suggest that UBA6^D^ T cells are involved in the development of multi-organ inflammation in mice. 

### 3.4. UBA6 Expression in SLE Patient T Cells

**Figure 4:**
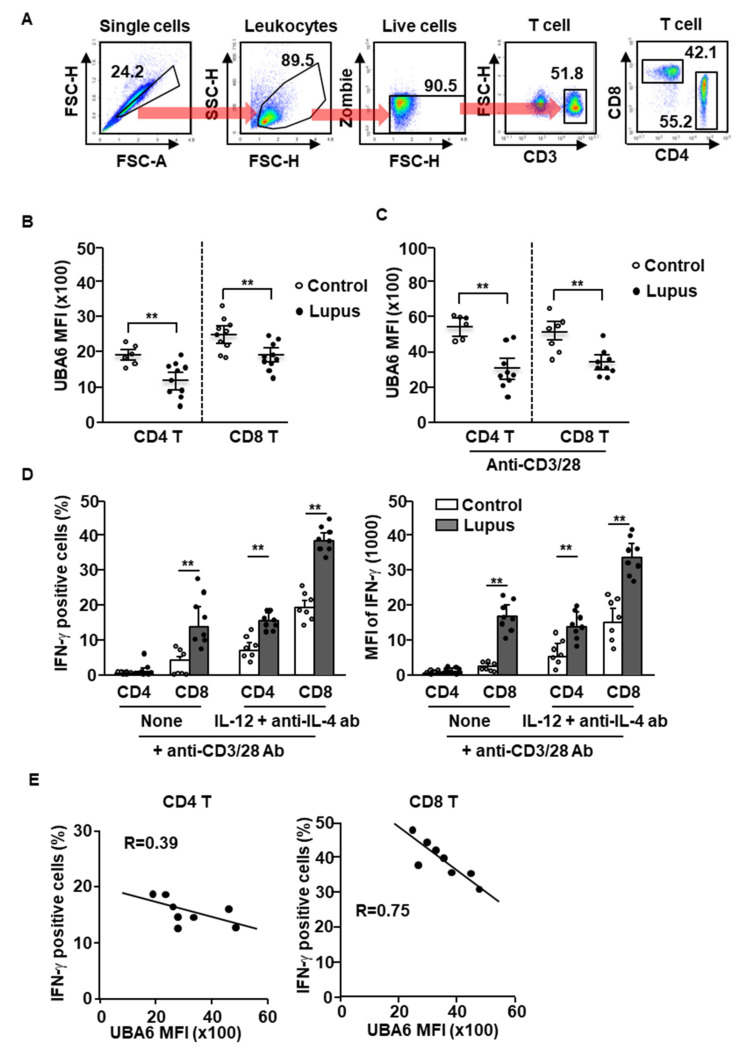
UBA6 negatively regulates IFN-γ production in SLE patient T cells. Peripheral blood mononuclear cells (PBMCs) from healthy and SLE donor analyzed expression levels of UBA6 in CD4 and CD8 T cells. (**A**) Definition of CD4 and CD8 T cells in PBMCs was shown. (**B**) MFI of UBA6 in healthy and SLE donor T cells were analyzed by flow cytometry (*n* = 6 to 10, two-way ANOVA, mean ± SEM, ** *p* < 0.01). (**C**) The isolated CD4 and CD8 T cells were stimulated with anti-CD3/28 and levels of UBA6 in the T cells were measured (*n* = 6 to 9, two-way ANOVA, mean ± SEM, ** p < 0.01). (**D**) Intracellular production levels (left panel) and MFI of IFN-γ (right panel) were analyzed in CD4 and CD8 T cells after stimulation with cytokine and mAbs (*n* = 7 to 8, two-way ANOVA, mean ± SEM, ** p < 0.01). (**E**) Co-relation of UBA6 expression and IFN-γ-production levels in SLE T cells were analyzed.

Given UBA6^D^ T cell-induced exacerbation of multi-organ inflammation in mice, we next compared the expression levels of UBA6 in healthy and SLE patient T cells. T cells of human peripheral blood were defined as CD3^+^CD4^+^ or CD3^+^CD8^+^ cells in live leukocytes, as shown in Figure 4A. The levels of UBA6 in the CD4 or CD8 T cells were significantly lower in patients with SLE than in healthy controls (Figure 4B). Next, we compared the change of UBA6 expression levels in healthy donor and SLE patient T cells by stimulation of anti-CD3/28 Abs and found that the increased levels of UBA6 were much lower in SLE patient T cells than in the T cells of healthy donors (Figure 4C). As expected, the T cells from patients with lupus showed hyperproduction of intracellular IFN-γ in response to cytokine and Ab stimulation (Figure 4D). Interestingly, hyperproduction of IFN-γ was negatively related to UBA6 expression in CD4 and CD8 T cells of patients with SLE (Figure 4E). Thus, these data indicated that UBA6 might negatively regulate production of IFN-γ in SLE patient CD4 and CD8 T cells. 

### 3.5. NF-κB Activation in UBA6D T Cells

TCR-mediated signaling pathways activate NF-κB and MAP kinases in T cells [18]. UBA6 is reported to be involved in controlling the activation of MAP kinases [9,10]; therefore, we next examined MAP kinase and NF-κB activation in T cells by western blot analysis. Before examining signaling activation, we measured alteration of TCR-β levels in UBA6^D^ T cells, given that degradation of TCR-β downregulates the signaling pathway. As shown in Supplementary Figure S3A, the stimulation of T cells by anti-CD3 Abs did not alter the expression levels of TCR-β in both control and UBA6^D^ T cells. Additionally, TCR-derived calcium flux levels were similar in both control and UBA6^D^ T cells (Supplementary Figure S3B). These data indicated that UBA6 does not alter proximal TCR signaling. 

**Figure 5:**
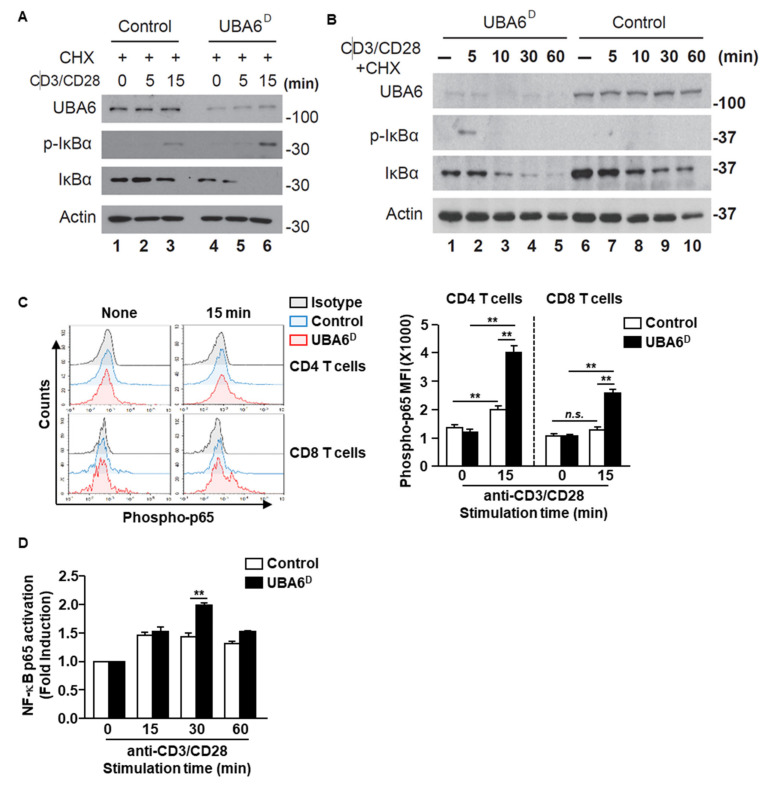
UBA6 is required for the inhibition of NF-κB activation in CD8^+^ T cells. Sorted naïve CD8^+^ T cells were activated with soluble anti-CD3 and anti-CD28 Ab for the indicated times. (**A**,**B**) Cells were activated in the presence of 50 μM cycloheximide. Whole cell lysates were prepared and analyzed by western blot analysis using p-IκBα, IκBα, and UBA6 Abs to detect these proteins after (**A**) shot time and (**B**) long time stimulation with anti-CD3 and anti-CD28 Ab. (**C**) Flow cytometry analyses of phospho-p65 the levels in CD4 and CD8 T cells. Cells were unstimulated (left) or stimulated for 15 min (right) (left panel). MFI of phosphor-p65 levels are shown (right panel) (*n* = 4, two-way ANOVA, mean ± SEM, ** p < 0.01). (**D**) Nuclear transcription factor NF-κB p65 activity was measured by ELISA-based TransAM NF-κB p65 kit (Active Motif) according to the manufacturer’s protocol. Data are representative of three independent experiments.

We next assessed TCR-mediated phosphorylation and degradation of IκBα, both of which are known to be prerequisites for NF-κB activation. Control-naïve CD3 T cells displayed IκBα phosphorylation in response to anti-CD3/CD28 Ab stimulation at 15 min with and without cycloheximide (Figure 5A and Supplementary Figure S4A). However, TCR-mediated IκBα phosphorylation markedly increased in UBA6^D^ CD3 T cells with and without cycloheximide (Figure 5A and Supplementary Figure S4), with detection extending to 5 min after stimulation. Although TCR stimulation led to reduced protein levels of IκBα in control T cells, IκBα degradation was significantly quicker in UBA6^D^ T cells. The acceleration of IκBα degradation/phosphorylation indicates increased NF-κB activation in UBA6^D^ T cells. Accordingly, UBA6^D^ CD4 and CD8 T cells showed enhanced phosphorylation of NF-κB p65 from 15 min after anti-CD3/28 Ab stimulation (Figure 5C). We also confirmed higher NF-κB p65 activity in UBA6^D^ T cells through an ELISA-based nuclear transcription factor activity assay and western blotting analysis (Figure 5D and Supplementary Figure S5). However, we did not detect enhanced phosphorylation of Erk1/2 or phosphorylation of c-Jun (Supplementary Figure S6A,B). Furthermore, we also analyzed the unfolded protein response in T cells and depletion of UBA6 in T cells did not alter the UPR proteins (Supplementary Figure S7). Overall, these results demonstrate that UBA6 controls the early phase of NF-κB activation in TCR-mediated signal transduction, thereby modulating T cell activation.

## 4. Discussion

This study demonstrates the important role of UBA6 in controlling IFN-γ production in CD4 and CD8 T cells. UBA6 deficiency in T cells resulted in the hyperactivity of IFN-γ production in both CD4 and CD8 T cells, contributing to exacerbation of multi-organ inflammation in RAG1-KO mice. Moreover, UBA6 levels were considerably lower in T cells from patients with SLE compared with those of healthy donors, which negatively correlated with IFN-γ production in T cells in response to stimulation. In the mechanism study of UBA6 in T cell activation, deficiency of UBA6 in T cells led to increased phosphorylation and degradation of IκBα, which led to increased activation of NF-κB p65. Our results provide insight into the role of UBA6 in T cell biology. Our work has identified the important role of UBA6 in T cells and demonstrated UBA6-mediated protein degradation as a mechanism for the regulation of IFN-γ production and exacerbation of autoimmune diseases.

Although some E3 ligases are reported to be critical to maintain peripheral T cell tolerance and their deficiency results in severe autoimmunity, we did not observe any sign of spontaneous autoimmune disease development in UBA6 T cell deficient mice. This may be attributed to the maintenance of functionally active mice (Supplementary Figure S2). Therefore, naïve T cells transferred to RAG1-KO mice without regulatory T cells developed multi-organ inflammation, likely reflecting that these E3s use different E2s under the control of charging by E1 enzyme. However, like Cbl-b, Itch, and Peli1-deficient T cells, UBA6 deficiency in T cells leads to their hyperactivation, suggesting that both the canonical UBA1 pathway and the non-canonical UBA6 pathway function within the immune system. 

SLE is associated with aberrant T cell function, dysfunction of T cell subsets, and imbalance of helper and regulatory T cells. As the systemic autoimmune process of mice lacking UBA6 is similar to that of patients with SLE, we suspected that dysfunction in the ubiquitin pathway is likely related to the pathogenesis of SLE. Elevated expression of IFN is present in SLE, and the IFN signature is the most prevalent molecular pathway activated in SLE. Additionally, the transcription factor NF-κB, which is activated by IFN and regulates IFN genes [19], is reportedly involved in SLE [20]. Our current study found that UBA6 expression markedly decreased in peripheral blood samples of patients with SLE compared with that of healthy controls. Patients with lupus also had increased expression of IFN-γ positive cells, and UBA6 expression was negatively correlated with IFN-γ positive cells. Patients with lupus express higher levels of NF-κB than healthy controls. Our result suggests that the known aberrant T cell subsets in SLE are likely to be partially related to dysfunction or deficiency of UBA6 due to its decreased T cell regulatory function. 

TCR stimulation by antigen recognition induces transcription factors on T cells, including NF-κB, nuclear factor of activated T cells (NF-AT), and activator protein 1 (AP-1). Activation of these transcription factors involves proliferation and activation of T cells and their differentiation to Th1, Th2, and Th17 cells. UBA6-deficiency promoted over activation of NF-κB, the primary transcription factor for Th1 and Th17 differentiation. However, UBA6 is only upregulated during differentiation of Th1, but not Th2 and Th17 cells, indicating that UBA6 may control the tolerance of Th1 immune responses by suppression of NF-κB activation. In addition, previous work has shown that deletion of UBA6 in other cell types such as fibroblasts inhibits MAPK (Erk1/2 and JNK1/2) activation [10]. In contrast, our data showed that deletion of UBA6 in T cells did not affect MAPK activation. Thus, the requirement for UBA6 in the MAPK and NF-κB pathways may depend on the cell type and stimuli. 

NF-κB signaling is well established as a critical step in many biological processes in the immune system, including inflammation, immunity, cell survival [14,21], and immune cell development [22]. Moreover, NF-κB is a central regulator of T cell survival, proliferation, and effector function during T cell activation. Although NF-κB activation in CD4^+^ T cells has been studied extensively, little is understood about its signaling in CD8^+^ T cells. It has been reported that certain modifications (phosphorylation of Ser311 and acetylation of Lys310) of NF-κB p65 are defective in anergic CD8 T cells, whereas early NF-κB activation events including IκB degradation and NF-κB nuclear translocation occur normally [23]. In addition, unlike CD4^+^ T cells, in which TCR downstream molecules (PKCθ, Bcl10, and Malt1) are essential for TCR-induced NF-κB activation, CD8^+^ T cells possess an alternative NF-κB activation that is independent of PKCθ, Bcl10, and Malt1 [24]. This study found that UBA6 is a novel negative regulator of NF-κB activation in CD3 T cells. Further work is required to determine the precise molecular mechanisms underlying NF-κB activation by UBA6, and to examine whether USE1 functions together with UBA6 in this context. Nevertheless, our study indicates that both the canonical and noncanonical arms of the UPS system function separately, and likely cooperatively, to modulate the immune system.

## Supplementary Materials

The following supporting information can be downloaded at: https://www.mdpi.com/article/10.3390/cells11010105/s1, Figure S1: Conditional deletion of UBA6 increased production of IFN-γ in CD4 and CD8 T cells; Figure S2: Regulatory T cells in UBA-deficiency mice; Figure S3: UBA6 deficiency does not alter downstream TCR signaling; Figure S4: UBA6 deficiency does not alter unfolded protein response pathway; Figure S5: UBA6 deficiency has increased p65 phosphorylation; Figure S6: Sorted naïve CD8 T cells were activated with anti-CD3/CD28 for the indicated time points; Figure S7: UBA6 deficiency does not alter unfolded protein response pathway.
