# Supplementary material for: Ubiquitin Activating Enzyme UBA6 Regulates Th1 and Tc1 Cell Differentiation
Source: Cells. 2021 Dec 29;11(1):105. doi: 10.3390/cells11010105 (PMC8750584; doi:10.3390/cells11010105)
Supplement: Supplementary file 1 [file cells-11-00105-s001.zip › cells-1462182-supplementary.pdf]

Supplementary Materials for

Ubiquitin-activating enzyme UBA6 regulates Th1 and Tc1 cell differentiation

Ji Yeon Lee *et. al.,*

Figure S1

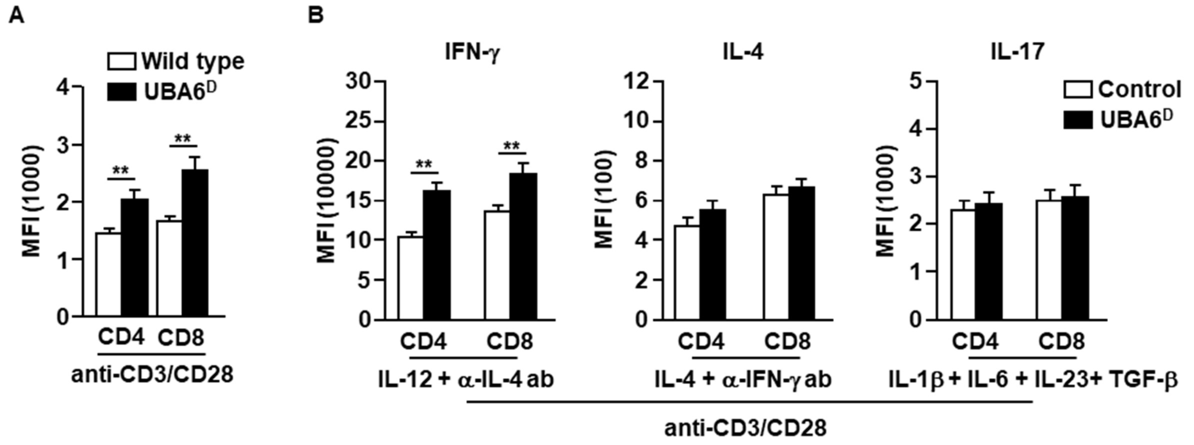

Figure S1. Conditional deletion of UBA6 increased production of IFN-γ in CD4 and CD8 T cells. (A) Mean fluorescence intensity (MFI) of IFN-γ in the CD4 or CD8 T cells were measured after stimulation with anti-CD3/28 Ab (n = 6 mice, two-way ANOVA, mean ± SEM). (B) MFI of indicated cytokines were analyzed after treatment with cytokine and mAbs (n = 6, two-way ANOVA, mean ± SEM).

Figure S2

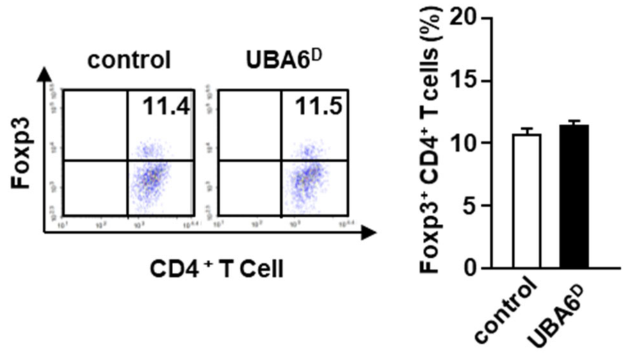

Figure S2. Regulatory T cells in UBA-deficiency mice. Frequency of regulatory T cells in UBA6^D mice were analyzed by intranuclear staining with Foxp3. (n = 5, one-way ANOVA, mean ± SEM).

Figure S3

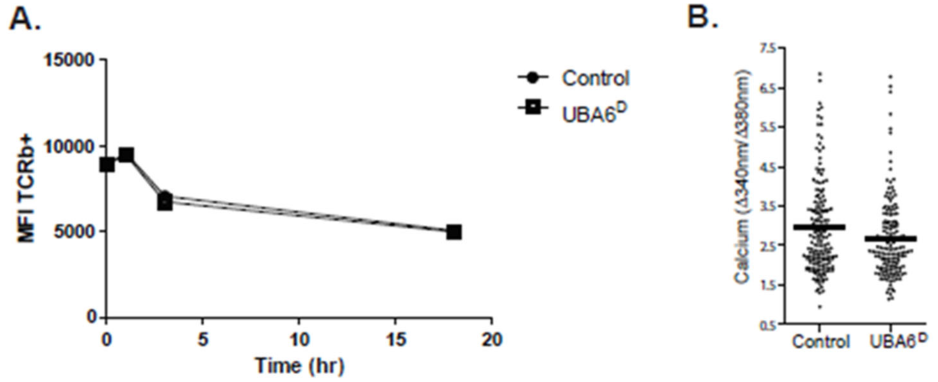

Figure S3. UBA6 deficiency does not alter downstream TCR signaling. (A) Naïve CD8 T cells were stimulated with anti-CD3 for the indicated time periods and analyzed for cell surface TCRβ expression. The mean fluorescence intensity of TCR-downmodulation is shown. T cells were pretreated with cycloheximide (50 μM) for prevention of new protein synthesis. Data are representative of two independent experiments. (B) Sorted naïve CD8 T from control and $UBA6^D$ mice were labeled with Fura-2 and stimulated on anti-CD3 and anti-CD28 coated plates. Calcium flux was assessed by microscopy at 8 min after addition of cells to coated plates. Each symbol indicates one individual cell. Data are pooled from triplicate samples per group and are representative of two independent experiments.

Figure S4

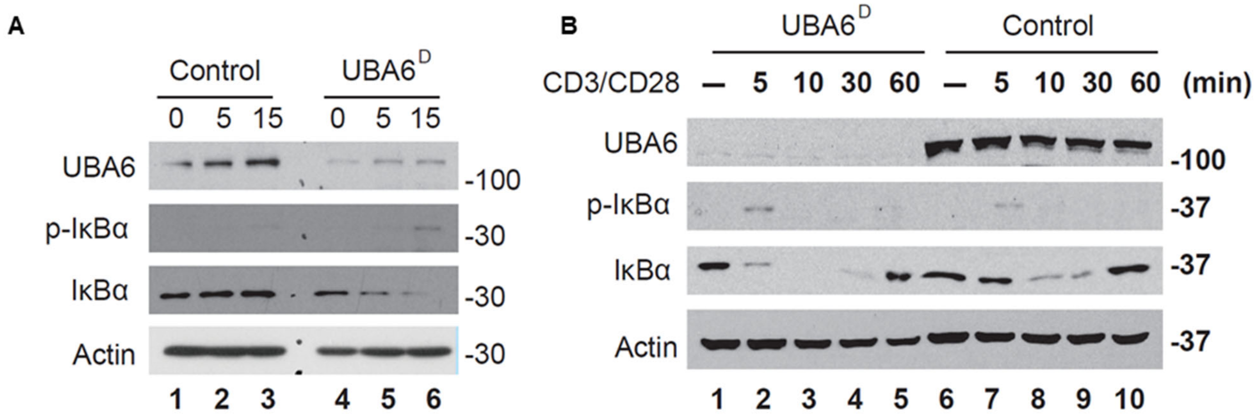

Figure S4. UBA6 is required for the inhibition of NF-κB activation in CD8+ T cells. Sorted naïve CD8+ T cells were activated with soluble anti-CD3 and anti-CD28 Ab for the indicated times. (A and B). Whole cell lysates were prepared and analyzed by western blot analysis using p-IκBα, IκBα, and UBA6 Abs to detect these proteins after (A) shot time and (B) long time stimulation with anti-CD3 and anti-CD28 Ab.

Figure S5

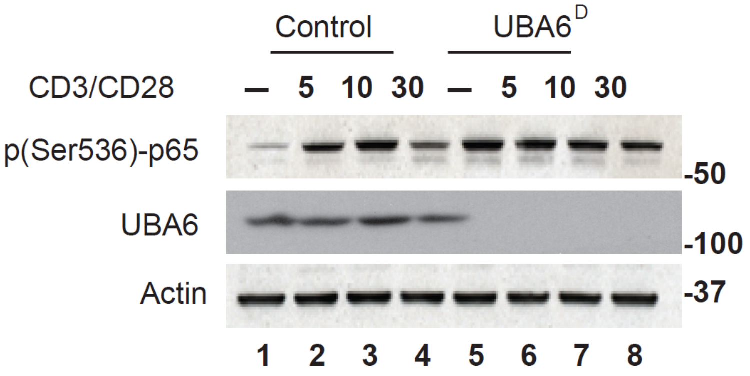

Figure S5. UBA6 deficiency has increased p65 phosphorylation. Sorted T cells were activated with soluble anti-CD3 and anti-CD28 Ab for the indicated times. Western blot analyses of phospho-p65 the levels in CD4 and CD8 T cells.

Figure S6

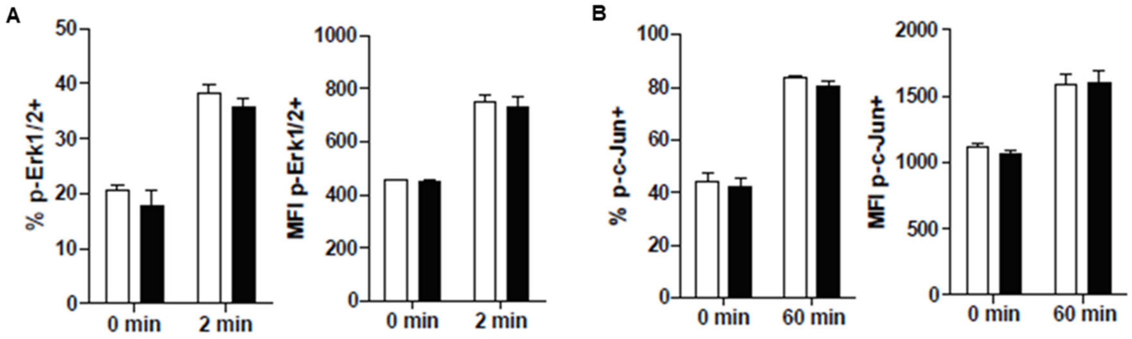

Figure S6. Sorted naïve CD8 T cells were activated with anti-CD3/CD28 for the indicated time points. Cells were fixed and evaluated by intracellular staining of phosphorylated Erk1/2 (A) and phosphorylated c-Jun (B). Percentage (left) and MFI (right) are shown. Data are representative of two independent experiments with 2-4 mice/group/experiment.

Figure S7

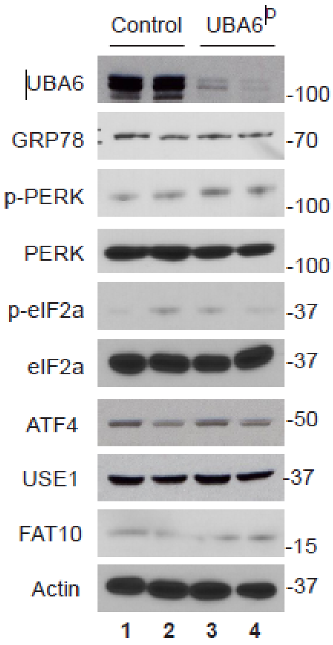

Figure S7. UBA6 deficiency does not alter unfolded protein response pathway.

Whole cell lysates were prepared and analyzed by western blot analysis using GRP78, p-PERK, PERK, p-eIF2a, eIF2, ATF4, USE1, FAT10 and Actin abs to detect these proteins.
